# Multiple environmental stressors induce complex transcriptomic responses indicative of phenotypic outcomes in Western fence lizard

**DOI:** 10.1186/s12864-018-5270-0

**Published:** 2018-12-05

**Authors:** Kurt A. Gust, Vijender Chaitankar, Preetam Ghosh, Mitchell S. Wilbanks, Xianfeng Chen, Natalie D. Barker, Don Pham, Leona D. Scanlan, Arun Rawat, Larry G. Talent, Michael J. Quinn, Christopher D. Vulpe, Mohamed O. Elasri, Mark S. Johnson, Edward J. Perkins, Craig A. McFarland

**Affiliations:** 1 U.S. Army Engineer Research and Development Center, Environmental Laboratory, Vicksburg, MS 39180 USA; 2 National Institute of Health - National Heart, Lung, and Blood Institute, Bethesda, MD 20892 USA; 3 Virginia Commonwealth University, School of Engineering, Richmond, VA 23284 USA; 4 IFXworks LLC, 2915 Columbia Pike, Arlington, VA 22204 USA; 5 Bennett Aerospace, Cary, NC 27511 USA; 6 Department of Nutritional Sciences and Toxicology, University of California Berkeley, Berkeley, CA 94720 USA; 7 Carlsbad Unified School District, Carlsbad, CA 92009 USA; 8 Department of Pesticide Regulation, California Environmental Protection Agency, Sacramento, CA 95812 USA; 9 Sidra Medicine, Education City (North Campus), Doha, 26999 Qatar; 10 Department of Natural Resource Ecology and Management, Oklahoma State University, Stillwater, OK 74078 USA; 11 U.S. Army Public Health Center, Aberdeen Proving Ground, Aberdeen, MD 21010 USA; 12 College of Veterinary Medicine, University of Florida, Gainesville, FL 32610 USA; 13 Department of Biological Sciences, University of Southern Mississippi, Hattiesburg, MS 39406-5018 USA

**Keywords:** Multiple stressors, Reptiles, Genomics, Munitions, Malaria, Food limitation, Climate change, TNT, Immune response

## Abstract

**Background:**

The health and resilience of species in natural environments is increasingly challenged by complex anthropogenic stressor combinations including climate change, habitat encroachment, and chemical contamination. To better understand impacts of these stressors we examined the individual- and combined-stressor impacts of malaria infection, food limitation, and 2,4,6-trinitrotoluene (TNT) exposures on gene expression in livers of Western fence lizards (WFL, *Sceloporus occidentalis*) using custom WFL transcriptome-based microarrays.

**Results:**

Computational analysis including annotation enrichment and correlation analysis identified putative functional mechanisms linking transcript expression and toxicological phenotypes. TNT exposure increased transcript expression for genes involved in erythropoiesis, potentially in response to TNT-induced anemia and/or methemoglobinemia and caused dose-specific effects on genes involved in lipid and overall energy metabolism consistent with a hormesis response of growth stimulation at low doses and adverse decreases in lizard growth at high doses. Functional enrichment results were indicative of inhibited potential for lipid mobilization and catabolism in TNT exposures which corresponded with increased inguinal fat weights and was suggestive of a decreased overall energy budget. Malaria infection elicited enriched expression of multiple immune-related functions likely corresponding to increased white blood cell (WBC) counts. Food limitation alone enriched functions related to cellular energy production and decreased expression of immune responses consistent with a decrease in WBC levels.

**Conclusions:**

Despite these findings, the lizards demonstrated immune resilience to malaria infection under food limitation with transcriptional results indicating a fully competent immune response to malaria, even under bio-energetic constraints. Interestingly, both TNT and malaria individually increased transcriptional expression of immune-related genes and increased overall WBC concentrations in blood; responses that were retained in the TNT x malaria combined exposure. The results demonstrate complex and sometimes unexpected responses to multiple stressors where the lizards displayed remarkable resiliency to the stressor combinations investigated.

**Electronic supplementary material:**

The online version of this article (10.1186/s12864-018-5270-0) contains supplementary material, which is available to authorized users.

## Background

Multiple anthropogenic pressures on natural environments are forcing complex stressor scenarios that may threaten the health and resilience of native species. As summarized in McFarland et al. [1], stressors that are characteristic of habitat encroachment, climate change, and chemical contamination are pervasive in western North America and result in concern for endemic reptilian populations. For example, increasing regional temperatures [2] threaten to expand disease vector ranges thereby increasing malaria infections in lizards [3] and reducing reproductive fitness [4, 5]. In concert with climate change, increased human immigration in the American West (http://www.census.gov/prod/cen2010/briefs/c2010br-01.pdf) is expanding habitat encroachment and reducing basic environmental resources for reptiles. As a result, extensive tracks of largely undeveloped military land in the American West are becoming the last remaining intact habitats in the ecoregion and thus serve as refugia for reptiles and other species. Military lands also pose unique challenges including munitions constituent (MC) contamination such as 2,4,6-trinitrotoluene (TNT) and 1,3,5-trinitro-1,3,5-triazine (RDX) on live fire training ranges, where exposures to these contaminants in laboratory studies have been observed to induce toxic responses in reptiles [1, 6]. The complex nature of these stressor exposures is a concern for sustaining viable endemic reptile populations, the failure of which could jeopardize range access for military operations and training. Given these challenges, we sought to experimentally evaluate the impact of multiple stressors on the health of a representative Western American reptile species, the Western fence lizard (WFL, *Sceloporus occidentalis*). Global transcriptomic expression was employed to uncover functional responses underlying critical phenotypic outcomes.

The present study leverages results from liver tissues collected in a previously reported study [1], which assessed in a pair-wise manner the effects of three representative stressors: (A) TNT dosing, (B) food limitation, and (C) malaria infection. Briefly, TNT dosing caused anemia, which was previously observed in WFLs [6] and across mammalian species [7]. TNT also elicited hormesis where body weights were increased relative to controls at low doses, but decreased at high doses [1]. Interestingly, TNT-induced hormesis was abolished when lizards were food-limited [1]. Food limitation decreased white blood cell (WBC) concentrations in the lizards [1], a characteristic response in malnutrition [8] possibly due to the energetic expense of maintaining basal immunity [9]. Malaria infection induced WBC activation including increased lymphocytes and monocytes, a response that was maintained regardless of food limitation [1]. Finally, both TNT exposure and malaria infection each tended to increase WBC concentrations, and TNT exposure may have enhanced the immune response to malaria [1]. These findings and additional responses were investigated as phenotypes of interest in global transcriptomics assays performed to identify potential causative mechanisms for individual stressor responses and stressor interactions.

Global transcriptomic expression assays are useful for establishing mechanistic and systems-level toxicological outcomes [10], which we have demonstrated for multiple munitions in an array of species [11–18]. To enable the transcriptomic investigation for *S. occidentalis,* we generated the first transcriptome for this reptile species and corresponding custom microarray tools for expression measurement. Differential-expression analyses were conducted to investigate the effects of individual TNT, food limitation, and malaria infection exposures in addition to all pairwise-stressor combinations in liver tissue. Pathway and annotation enrichment analysis [19] provided functional-genomic responses which were integrated into a correlation analysis to associate expression with toxicological phenotype data derived from McFarland et al. [1]. This work was conducted to test the hypothesis that multiple ecosystem-level stressors characteristic of habitat degradation and climate change have no interactive effects on lizard health. Overall, the results revealed complex and sometimes unexpected responses to multiple stressors. Lizards demonstrated remarkable resiliency for the stressor scenarios tested herein.

## Methods

Detailed methods for animal husbandry, exposure techniques, and clinical toxicology are described in McFarland et al. [1]. Bioassays conducted in that study provided the source tissue for all transcript-expression assays described herein. The original laboratory colony of WFLs maintained at Oklahoma State University was established from lizards collected from the San Joaquin Valley in Fresno and Tulare Counties, California, USA. Lizard exposures were conducted at the U.S. Army Public Health Center (USAPHC) using males obtained from generations F4 and F5. All animal-use protocols were conducted consistent with Good Laboratory Practices and approved by the Institutional Animal Care and Use Committee at the U.S. Army Public Health Center.

### Summary of stressor bioassays

Briefly, three 30-day experimental assays utilizing male lizards were conducted including: Exposure 1 - TNT x food limitation, Exposure 2 - food limitation x malaria infection, and Exposure 3 - TNT x malaria infection [1]. Each study incorporated a factorial treatment arrangement within a completely randomized experimental design. The malaria treatment included infected and non-infected control lizards. Food limitation treatments included an *ad libitum* control (fed 10 crickets daily) and at least one reduced food ration (fed 5 or 2 crickets daily). TNT exposures consisted of daily oral doses of corn oil as a control or a corn oil-TNT suspension at 5, 10, 20, or 40 mg/kg/day. All treatments included 10 biological replicates (10 individually caged lizards) per condition. At the completion of the exposures, animals were euthanized by CO_2_ asphyxia and dissected. Samples from brain, bone marrow, gut, heart, liver, and testes tissues were fixed in RNA Later™ (Ambion, Austin, TX) following manufacturers recommendations and stored at − 80 °C until RNA extraction. Blood and various tissues were also collected for clinical endpoint analysis where individual and pair-wise stressor effects were tested for 29 toxicological endpoints [1].

### Tissue fixation and RNA extraction

Tissue samples used to construct the normalized complimentary DNA (cDNA) libraries for WFL were collected from five control animals from each experiment. The RNA pool used to construct the cDNA library included RNA extracted from brain, bone marrow, gut, heart, liver, and testes. RNA extraction was conducted using RNeasy Mini RNA extraction kits (Qiagen Inc., Valencia, CA). RNA quality was assessed using an Agilent 2100 Bioanalyzer (Agilent Technologies, Waldbronn, Germany) with RNA 6000 Nano LabChips® RNA. Only samples with a 28 s/18 s ratio ≥ 2.0 and RNA integrity number (RIN) ≥7.0 were used for downstream applications.

### cDNA library construction and normalization

The RNA compilation used to construct the WFL cDNA library included 500 ng of total RNA from 46 total samples. The SMART™ PCR cDNA Synthesis Kit (Clonetech Laboratories Inc. Mountain View, CA) was utilized to reverse-transcribe 1.0 μg of the RNA compilation sample into full length cDNAs. The cDNA libraries were normalized prior to sequencing to capture both high and low abundance transcripts using the Trimmer cDNA Normalization Kit (Evrogen JSC, Moscow, Russia).

### cDNA sequencing

The normalized cDNA library was sequenced using a 454 Life Sciences GS-FLX sequencer (Roche / 454 Life Sciences, Branford, CT, USA) utilizing a protocol to resolve 400 base-pair (bp) reads. Briefly, cDNAs were nebulized and size-selected for 500 to 800 bp fragments. Two primer sequences, Adaptor A and Adaptor B, were ligated to the fragments. cDNAs containing both an A and a B adaptor were melted into single stranded DNA, immobilized onto DNA capture beads and emulsified in oil for polymerase chain reaction (emPCR). The emPCR was titrated to determine the optimal amount of single stranded DNA (ssDNA) needed to create a 1:1 DNA fragment to bead ratio. emPCR was performed and the amplified library was loaded onto a 70 × 75 mm PicoTiterPlate (Roche / 454 Life Sciences) and sequenced for one full plate run.

### Sequence processing and annotation

The genome-scale transcriptome was used for EST-based clustering and assembly via The Gene Indices Clustering Tools (TGICL) [20], which uses mega-basic local alignment search tool (megaBLAST) [21] for homology-based clustering and contiguous sequence (contig) assembly program 3 (CAP3) [22] for assembly. Unigenes consisting of contigs and/or single sequences (singlets) longer than 200 bp were selected for BLASTx (protein) analysis [21] for homology-based coding-region similarity detection and annotation against five sets of publicly available protein sequence databases: National Center for Biotechnology Information (NCBI) NR.aa, Refseq, and European Bioinformatics Institute (EBI) UniProt-SwissProt, Uniref90, and Uniref100. The central processing unit intensive computational biology analysis pipelines of clustering, assembly, and annotation were run via Portable Batch System (PBS) (http://pbspro.org/) through the Department of Defense (DoD) supercomputers Diamond (SGI Altrix ICE) and Jade (Cray XT4).

### Microarray design

All contig and singlet sequences with significant BLASTx matches (E > 10^− 5^) were submitted to eArray (https://earray.chem.agilent.com/earray/, Agilent Technologies) for microarray probe development where two unique probes were generated for each transcript sequence target. All probes flagged for potential cross-hybridization to another probe were removed from the probe set. The remaining probes were sorted by the E-value corresponding to each target sequence and the 30 K probes with the lowest E-value scores were printed on the microarray in duplicate for a total of 60 K probes per microarray. The Agilent 8 × 60 K, 2 μm feature size microarray platform was used for transcript expression assays (Agilent Technologies).

### Microarray hybridizations

**Fig. 1:**
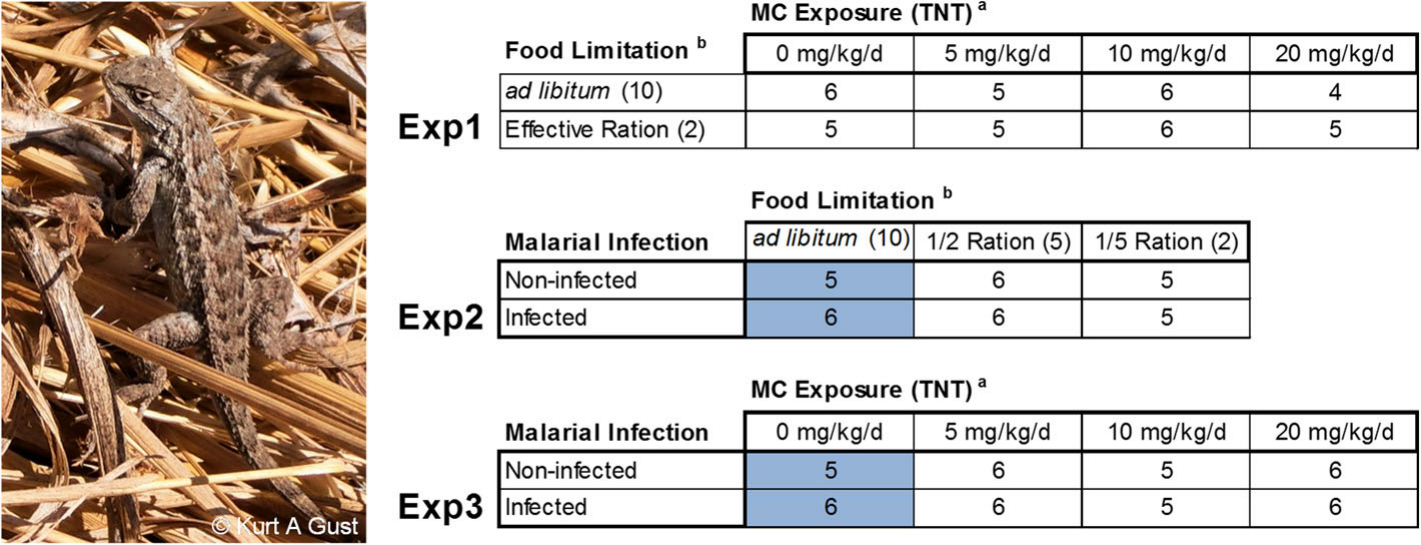
Experimental designs for microarray experiments across the three single/combined-stressor exposures in the Western fence lizard (*Sceloporus occidentalis*). Numbers within each cell represent the number of biological replicate microarray hybridizations contributing to the overall microarray analysis in the exposure matrix. Cells highlighted in gray represent microarrays hybridized using the same biological replicates for corresponding treatments across Exposure 2 and Exposure 3. Exp1, Exp2 and Exp3 represent Exposure 1, Exposure 2 and Exposure 3, respectively

The Agilent One-Color Microarray Hybridization protocol (Agilent Technologies) was utilized for microarray hybridizations following manufacturer’s recommendations and 500 ng of total RNA extracted from liver tissues in each individual lizard replicate. Six replicates were hybridized to microarrays for each treatment combination represented in the three stressor-combination exposures. The six replicates were selected at random from the 10 total lizards per treatment from McFarland et al. [1] using a random number generator. Randomized block designs were utilized to eliminate the potential for batch effects among two temporally separated microarray hybridization events. Three of the six total biological replicates for each treatment were randomly assigned to one of two blocks using a random number generator. Some microarray hybridizations were compromised and were not included in the microarray analysis, however all treatments included a minimum of four replicates where all but one treatment included five or six replicates total (Fig. 1).

### Microarray analysis

An Agilent Technologies, High-Resolution Microarray Scanner (Model G2505C, Agilent Technologies, Santa Clara, CA, USA) was used to scan microarray images at 2 μm resolution. Data were extracted using Agilent Feature Extraction software, version 9.5.1 (Agilent Technologies). Internal control spots were analyzed to confirm that signal data was within the linear range of detection. GeneSpring version GX 11.0.2 (Agilent Technologies) was used to normalize data using quantile normalization followed by baseline transformation to the median of all samples. Statistical analysis was performed using GeneSpring where two-way ANOVA (*p* ≤ 0.05) with Benjamini-Hochberg multiple-testing correction and 1.5 fold-change cutoff was used to identify differentially expressed transcripts. Principal components analysis and hierarchical clustering analysis for transcripts with significant differential expression were conducted using GeneSpring and Multi-Experiment Viewer software version 4.9 [23], respectively.

### Annotation enrichment

The database for annotation, visualization and integrated discovery (DAVID), version 6, [19] was used to derive significantly enriched annotation clusters for gene-transcripts that had significant differential expression. The WFL annotations connected to the microarray served as the “background” transcriptome. The annotation clusters included gene ontology, canonical pathway and protein motif annotations, which provided an integrated suite of classifications within each significant cluster. Annotation cluster enrichment was calculated as the geometric mean of all the enrichment *P*-values (Expression Analysis Systematic Explorer, EASE scores) for each annotation term associated with the gene members in the group where higher composite enrichment scores represent greater enrichment of the cluster function [19]. The significant annotation clusters were used to posit categorical matches to summary exposure-responses as well as matches to the toxicological observed phenotypes first described in McFarland et al. [1].

### Expression – Phenotypes correlation analysis

Correlation analysis was employed as a means to integrate transcriptional gene expression data derived from the multiple-stressor exposures to the toxicological phenotypes observed in McFarland et al. [1]. The transcript expression and phenotype data were standardized (i.e. z-scores defined as ratio of difference with mean to standard deviation) before computing pairwise Pearson’s correlation metric. To correlate differentially expressed clusters with phenotypes, a representative eigen gene for each cluster, defined as the first principal component of each cluster was computed using the ‘*moduleEigengenes*’ function in weighted gene co-expression network analysis (WGCNA) R package [24].

### Reverse-transcriptase quantitative polymerase chain reaction (RT-qPCR)

Microarray results were validated using RT-qPCR of 41 transcripts of importance across the three exposures in addition to the 18S ribosomal RNA (rRNA) for Eastern fence lizard, *Sceloporus undulatus* (Additional file 1: Table S1) which was used as a control/housekeeping reference gene. DNase- (Qiagen, Valencia, CA, USA) treated total RNA from all biological replicates previously used in microarray hybridizations were examined by RT-qPCR. Briefly, 500 ng of total RNA was reverse transcribed into cDNA in a 6.3 μL reaction containing 250 ng of random primers and SuperScript III reverse transcriptase (Invitrogen, Carlsbad, CA), following the manufacturer’s protocol. RT-qPCR was performed using an Applied Biosystems (ABI) Prism 7900HT Sequence Detection System (Applied Biosystems, Foster City, CA). Cycling parameters for cDNA amplification were 95 °C for 15 min, 40 cycles of 95 °C for 15 s, and 60 °C for 1 min. RT-qPCR data was analyzed with SDS 2.2 software package (Applied Biosystems, Foster City, CA) using the ΔΔCT method to quantify results as recommended by the developer. Primer melt curves were conducted after RT-qPCR. Primer sets that did not have a single conserved melt peak were discarded. Fold change values (log_2_) were calculated using relative quantification results where values represent transcript expression in stressor treatments relative to controls. The 95% confidence interval (95% C.I.) was calculated around the mean relative expression for each treatment. Confidence intervals that did not include unity were considered differentially expressed relative to controls [17]. Additionally, linear-regression analysis was conducted comparing transcript expression from microarray analysis versus RT-qPCR to determine correlations among expression assessment methods using SigmaPlot (v13.0, Systat Inc. San Jose, California).

## Results

### cDNA library sequencing, annotation and microarray

The sequencing effort produced over 328 million base calls for the WFL in 928,780 sequence reads. Average sequence read length for WFL was 354 bases. The sequence data set was clustered, assembled and annotated with protein-coding information. A total of 928,759 expressed sequence tags (ESTs) were generated from the 928,780 total DNA sequences after removing control sequences. In all, 53,897 contigs and 5065 singlets totaling 58,962 unigenes were identified. Among the unigenes, 33 to 44% of singlets and contigs were annotated for protein-coding potential via homology-based annotation against NCBI NR.aa and Refseq, and EMBL-EBI UniProt-SwissProt, Uniref90, and Uniref100 protein sequence reference databases (Additional file 1: Table S2). Significant annotation matches for Refseq (proteins) identified homologous matches to 175 different species with the most matches to the *Gallus* (5596), *Taeniopygia* (3197), and *Monodelphis* genuses (1814), and 184 orthologous matches to existing *S. occidentalis* RefSeq annotations (see RefSeq protein annotations in Additional file 1: Table S3).

### Microarray results overview

**Fig. 2:**
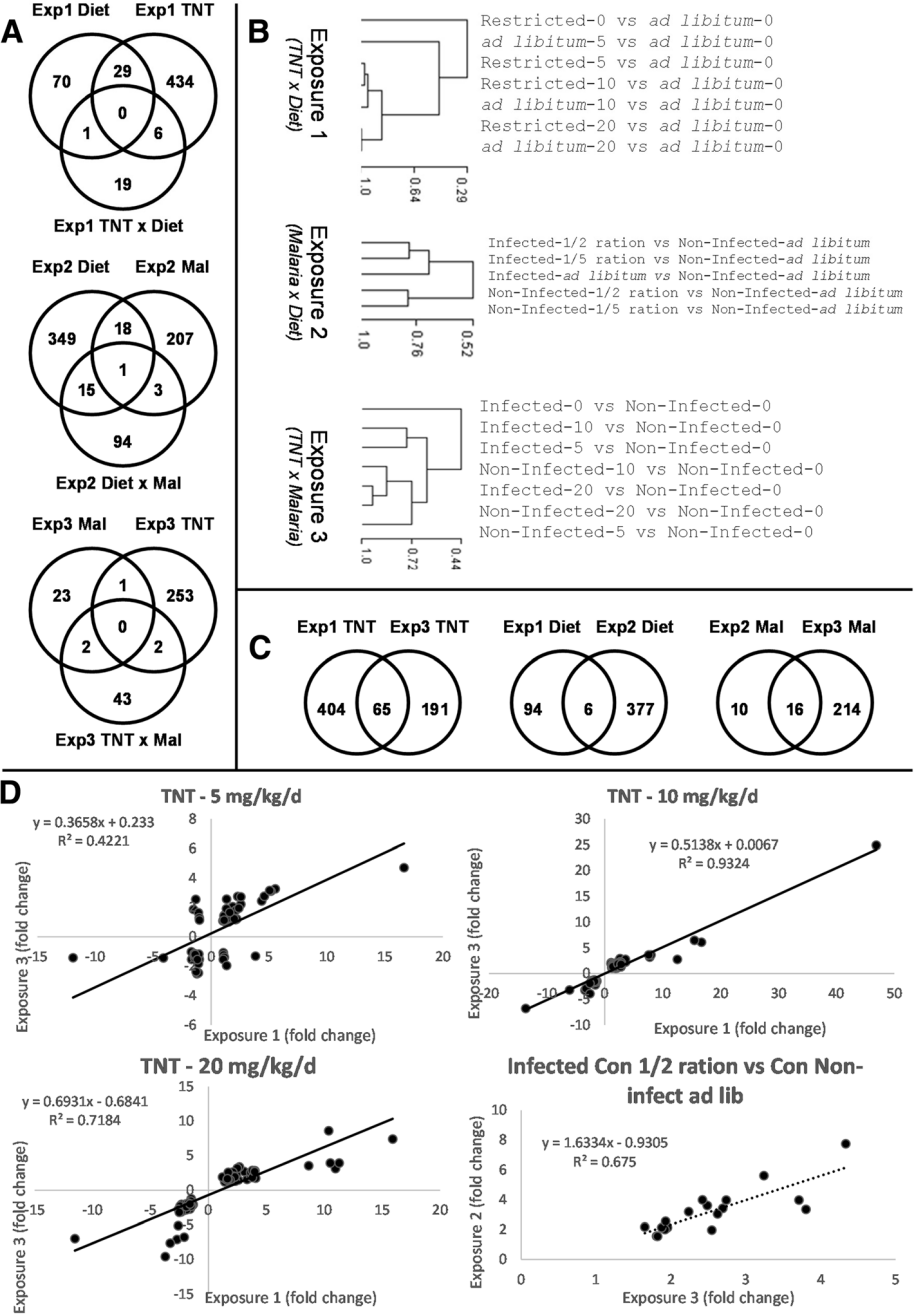
Summary results for microarray analyses. Venn diagrams in panel (**a**) display the differentially expressed transcripts found in common among treatments based on matching RefSeq Accession IDs. Hierarchical clustering analyses (**b**) identifed relationships based on significant differential transcript expression among treatment combinations for each exposure. Transcripts differentially expressed in common among repeated experimental exposures (**c**) and correlations in expression among these transcripts (**d**) provided opportunities for assessing reproducibility of experimental results. Note that no correlation was calculated for Diet given that only two targets were found in common. Key for Venn Diagrams: Exp = Exposures 1–3, Diet = food level, and Mal = malaria treatment. For hierarchical clustering analysis: infected = malaria infection, restricted = restricted diet and the values 0, 5, 10 and 20 = TNT dosing levels in mg/kg-d

Significant differential transcript expression was observed for individual stressors and in the pair-wise stressor combinations for all experimental exposures (Exposures 1–3, Additional file 1: Table S4A-C). The microarray data is available at the Gene Expression Omnibus website (GEO, ID# GSE116026: https://www.ncbi.nlm.nih.gov/geo/query/acc.cgi?acc=GSE116026). Differentially expressed transcript sets tended to be unique to each stressor type and for the pair-wise stressor combinations (Fig. 2a), and principal component analysis showed discrete separation by stressor type where replicates for each treatment-level tended to cluster together (Additional file 2: Figure S1). Hierarchical clustering analysis of differentially expressed transcripts indicated sorting by TNT dose regardless of the diet restriction treatment in Exposure 1, whereas expression sorted by malaria infection regardless of food restriction in Exposure 2, and in Exposure 3 expression for the malaria-only treatment sorted separately relative to all TNT exposures in malaria infected or non-infected states (Fig. 2b). Given the intentional semi-redundancy of the experimental design where each individual stressor (TNT exposure, food limitation and malaria infection) was investigated in two unique exposures (Fig. 1), we were also able to assess the reproducibility of expression patterns for each stressor. The conservation of differentially expressed transcript sets for each stressor among exposures were 6, 25 and 62% in food restriction, TNT exposure, and malaria infection treatments, respectively (Fig. 2c). Comparison of fold changes for transcripts differentially expressed in common among the repeated experimental trials demonstrated positive correlations further establishing reproducibility in the direction and magnitude of transcriptomic expression for the TNT and malaria exposures (Fig. 2d). Overall, the results of the repeated experimental trials for the individual food restriction, TNT dosing, and malaria infection treatments indicated that the general stressor (food restriction) generated a less repeatable transcriptomic response than the stressors with more specific mechanisms of action (TNT and malaria).

Statistical interactions among stressors were observed in all exposures where higher proportions of significant transcripts were observed for the malaria infection x diet interaction and the malaria x TNT interaction relative to the number found for TNT x diet (Fig. 2a). The transcripts differentially expressed in response to the stressor interactions tended to be unique relative to those affected by the individual stressors (Fig. 2a). Expression for these transcripts showed divergent patterns across stressor series where hierarchical clustering analysis of composite expression sets indicated the interaction effects outweighed the response of any single stressor (Additional file 2: Figure S2). In addition to transcripts identified by statistical interactions among stressors, binary effects combinations of individual stressors on transcriptional expression were also considered when developing the functional interpretations for combined-stressor effects (see Discussion).

### Annotation enrichment

**Fig. 3:**
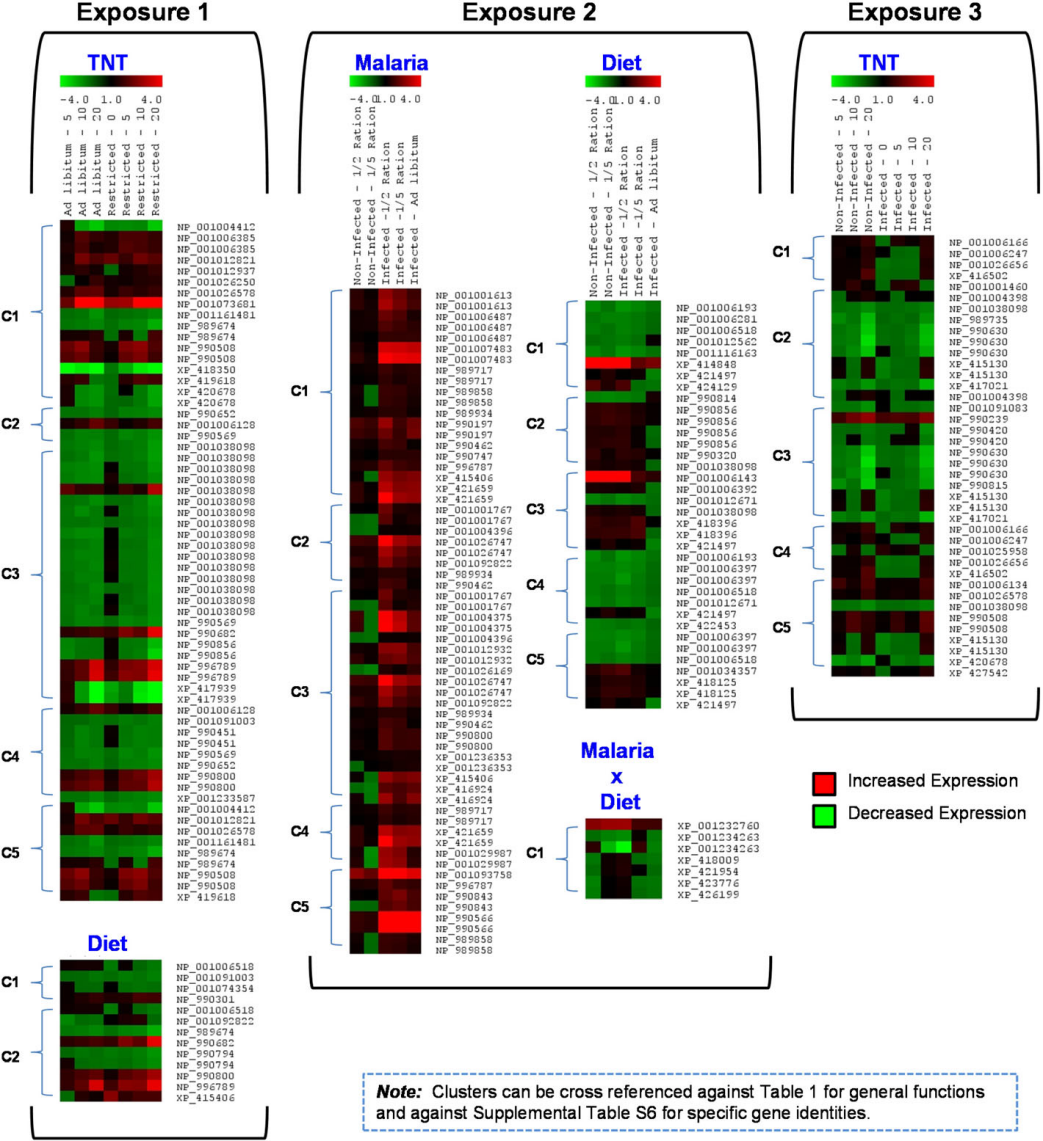
Transcript expression profiles for the significantly enriched annotation clusters (i.e. C1-C5). Significant clusters are mapped to each treatment for all exposures (See Fig. 1 for design). Clusters can be cross referenced against Table 1 for general functions and against Additional file 1: Table S6 for specific gene identities and expression values

Significantly enriched annotation clusters were examined across the three exposures to provide a functional characterization of treatment effects (Table 1 and Additional file 1: Tables S5 and S6). Examination of the most statistically significant enriched clusters within Exposure 1 indicated that TNT effected transcription of genes involved in proteolysis, carbohydrate biosynthesis, lipid metabolism, glycolysis, and hemostasis (Table 1). The food limitation treatment primarily affected genes involved in the mitochondrial cellular component and calcium and cation binding. The malaria infection treatment within Exposure 2 caused significantly increased expression of genes involved in a broad suite of immune responses including positive regulation of T cells, lymphocyte activation, leukocyte activation, immunoglobulin signaling and programmed cell death (Table 1, Fig. 3). Additionally, zinc finger binding elements that can act as immune response regulators [25] were enriched in the malaria infection treatment. As was observed in Exposure 1, food limitation in Exposure 2 caused significant enrichment of transcripts involved in mitochondrial function. Additionally, in Exposure 2, enrichment of endoplasmic reticulum membrane and organelle lumen space was observed along with effects on cofactor binding and plasma lipoproteins indicating changes in lipid metabolism (Table 1). The malaria infection x food limitation treatment interaction caused significant enrichment of zinc ion binding processes. Finally, in Exposure 3, TNT dosing caused enrichment of transcripts involved in proteolysis, as was observed in Exposure 1, in addition to enrichment of transcripts involved in ribonucleoprotein function, homeostatic processes, and vesicle-mediated transport. Curiously, no significantly enriched clusters were observed for the malaria infection treatment in Exposure 3, and there were no clusters in the TNT x malaria infection treatment interaction (Table 1). The TNT treatment tended to dominate the overall expression profile within Exposure 3 (Fig. 2c), thus it is likely that the malaria responses were masked by the response to TNT where the TNT insult received physiological priority in the multi-stressor complex. Transcript expression patterns for all top clusters are provided in Fig. 3, which can be cross referenced against Table 1 for general functions and against Additional file 1: Table S6 for specific gene identities, significance-level within treatments, and expression values. Trends included dose-responsive increases in expression within protein catabolism clusters and widespread increases in expression across multiple immune-related clusters.

**Table 1:** Statistically enriched annotation clusters for each stressor exposure in the Western fence lizard (*Sceloporus occidentalis*) where top clusters are categorically matched to functions and toxicological phenotypes

Exposure 1 (TNT x Diet)						
Treatments	Significant Annotation Clusters	Cluster ID	Enrichment Score	Top Clusters - Enriched Function (Summary of Cluster Components)	Categorical Match 1 - Enriched Function Summary Response	Categorical Match 2 - Cluster to Toxicological Phenotype
TNT	18	C1	2.14	proteolysis, peptidase activity, serine hydrolase activity	Protein Catabolism	↓: ALB, GLOB, TP, & TS
		C2	1.74	carbohydrate biosynthesis processes	Carbohydrate Storage	↓ Body Weight
		C3	1.55	lipid transport, lipid localization, lipoprotein metabolism, extracellular	Lipid Metabolism	↓ Body Weight, ↑ Liver Wt.
		C4	1.42	glycolysis, carbohydrate catabolic process, glucose catabolic process	Carbohydrate to Cellular Energy	↓ Body Weight; ↓ Feeding
		C5	1.26	hemostasis, blood coagulation, wound healing, endopeptidase activity	RBC Retention & Wound Healing	↓: Hb, Hct, RBC & MCHC; ↑ spleen wt.
Diet	2	C1	1.12	mitochondrion, mitochondrial envelope	Cellular energy production	↓ Body Weight; ↓ Inguinal fat
		C2	0.51	calcium binding, cation binding, metal binding	Cell signaling	–
INI x Diet	0	–	–	–		
Exposure 2 (Malaria Infection x Diet)						
Malaria	9	C1	2.57	positive regulation of T cell, lymphocyte & leukocyte activation. Regulation of immune system process, regulation of programmed cell death	Immune Response	↑: WBC, Lympho, Mono; ↓ H/L
		C2	1.42	zinc finger - RING-type, zinc finger C3HC4 RING-type	Immune Response	↑: WBC, Lympho, Mono; ↓ H/L
		C3	1.10	zinc finger - C2H2, cation binding	Immune Response	↑: WBC, Lympho, Mono; ↓ H/L
		C4	1.02	apoptosis, programmed cell death	Immune Response	↑ WBCs
		C5	0.60	immunoglobulin-like, signal peptide	Immune Response	↑ WBCs
Diet	10	C1	1.14	cofactor binding, coenzyme binding, oxidation reduction	Electron Iransport, Energy Production	↓ Feeding; ↓: WBC, Lympho, H/L
		C2	1.04	plasma lipoprotein particle, protein-lipid complex, extracellular space	Lipid Metabolism	↓ Feeding; ↓: WBC, Lympho, H/L
		C3	0.86	endoplasmic reticulum membrane, endomembrane system	Cellular & endomembrane transport	–
		C4	0.80	mitochondrion, mitochondrial envelope	Cellular energy production	↓ Feeding; ↓: WBC, Lympho, H/L
		C5	0.54	organelle lumen, membrane-enclosed lumen - Nested in Mitrochnodria	Cellular energy production	↓ Feeding; ↓: WBC, Lympho, H/L
Malaria x Diet	1	C1	0.71	zinc ion binding, cation binding	Immune Response	Malaria: ↑ WBCs & Lympho; Diet: ↓ WBCs & Lympho
Exposure 3 (TNT x Malaria Infection)						
TNT	10	C1	1.54	ribonucleoprotein complex biogenesis, rRNA processing	Ribosomal Function	–
		C2	1.49	homeostatic process, membrane fraction	PPAR Signaling, Lipid Metab.	↓ Body Weight, ↑ Liver Wt.
		C3	1.15	vesicle-mediated transport, endocytosis	Intracellular transport, Immune Sys.	↑ WBCs
		C4	1.11	WD40 repeat, RNA processing, ribonucleoprotein complex	Transcriptional Reg., Signal Trans.	–
		C5	0.85	proteolysis, protein catabolic process, endopeptidase activity	Protein Catabolism	↓ Body Weight
Malaria	0	–	–	–		
TNT x iviaiaria	0	–	–	–		

The top clusters column provides representative biological processes, molecular functions, cellular components, canonical pathways and functional motifs within each enriched cluster (See Additional file 1: Tables S5 and S6 for full detail). The categorical matches to enriched function represent a summary response of the enriched annotation cluster. The categorical matches to toxicological phenotypes provide putative connections to toxicological observations summarized in Table 2 of McFarland et al. [1]

Key for Toxicological Phenotype Symbols: *ALB* = Albumin, *GLOB* = Globulin, *TP* = Total Protein, *TS* = Total Solids, *Hb* = Hemoglobin Concentration, *Hct* = Hematocrit, *RBC* = Red Blood Cell Concentration, *MCHC* = Mean Corpuscular Hemoglobin Concentration, *WBC* = White Blood Cell Concentration, *Lympho* = Lymphocyte Concentration, *Mono* = Monocyte Concentration, *H/L* = Heterophyl / Lymphocyte Ratio

### Co-expressed transcripts and phenotypes

**Fig. 4:**
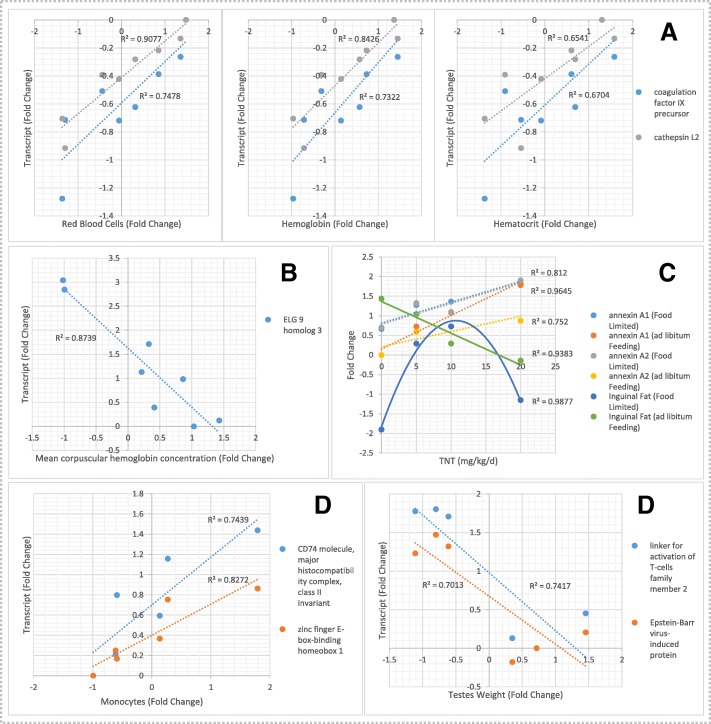
Relationships between differentially expressed gene transcripts identified within significant annotation clusters and toxicological phenotypes in the Western fence lizard. The plots represent selected relationships identified by correlation analysis (Additional file 1: Table S8) paired against expression of transcripts identified within significant annotation clusters (Additional file 1: Table S6). Toxicological phenotype data represented herein were significantly affected by the experimental exposures as described in McFarland et al. (2012) [1]

The complete set of highly correlated (Pearson − 0.85 > *r* > 0.85) differentially expressed transcripts and toxicological phenotypes characterized in McFarland et al. [1] are provided in Additional file 1: Table S7. The analysis identified gene-to-phenotype connections for a variety of transcripts including those contributing to significantly enriched annotation clusters (Additional file 1: Table S8). Specific observations including positive correlations between expression of coagulation factor IX (NP_989,674.1) and cathepsin (NP_001161481.1) with hematological parameters including red blood cell count, hemoglobin concentration and hematocrit in exposure 1 (Fig. 4a). Additionally, a negative correlation between expression of Egl-9 family hypoxia inducible factor 3 (EGLN3, XP_421,233.2) transcripts with mean corpuscular hemoglobin concentration (MCHC) was observed (Fig. 4b) and positive dose-response relationships between TNT concentration and annexins expression were observed, regardless of feeding level (Fig. 4c). In Exposure 2, enriched annotation clusters including immune responses (Table 1) were positively correlated with expression of immune-related genes including CD74 molecule, major histocompatibility complex, class II (NP_001001613.1) and zinc finger E-box-binding homeobox 1 (NP_990462.1) with increased monocytes in response to malaria infection (Fig. 4d). Conversely, increased transcriptional expression of immune related genes was negatively correlated with testes size (Fig. 4e) suggesting potential bioenergetics tradeoffs between immune response and growth and illustrating the potential for complex integrated responses to stressors.

### RT-qPCR results

RT-qPCR target selection was conducted to maximize coverage of affected functions by prioritizing gene transcripts included in significantly enriched annotation clusters and highly correlated gene-phenotype pairs identified by correlation analysis (Additional file 1: Table S9). RT-qPCR validated 80, 78 and 75% of transcripts identified to have significant differential expression in microarray assays for Exposure 1, 2 and 3, respectively (Additional file 1: Table S9). Linear regression indicated significant positive correlations in transcript expression between the microarray and qPCR expression results (*p* < 0.001 for all exposures and R^2^ = 0.78, 0.62 and 0.60 for Exposure 1, 2 and 3, respectively). The concordance in results among the transcript expression quantification methodologies across the three exposures indicates that the microarray assays provided reliable transcript-expression data for the mechanistic exposure-effect results described herein.

## Discussion

This research effort has provided the first transcriptome characterization for the reptile species, *Sceloporus occidentalis*. The transcriptome characterization provided the foundation to investigate global transcriptomic responses to stressors related to climate change, (malaria infection), habitat degradation (food limitation), chemical contamination (TNT exposure) and the binary combinations of each. Each stressor induced unique changes in transcript expression as well as unique transcript expression profiles in binary stressor exposures (Fig. 2a). Interestingly, expression patterns for stressors having specific effects (i.e., malaria infection and TNT exposure) tended to have better reproducibility compared to the nonspecific stressor (food limitation, Fig. 2b). Statistically enriched annotation clusters and correlation of significant transcript sets to toxicological phenotypes indicated potential for impacts on unique biological functions among stressor treatments (Table 1, Fig. 3 and Fig. 4). Functional interpretation of these results can provide important inferences to the mechanistic effects of the individual stressors and stressor combinations. Important putative gene-phenotype connections are summarized in Table 2. Key examples included decreased expression of transcripts coding for genes involved in lipid metabolism in TNT exposures (Table 1, Fig. 3, Additional file 1: Table S6), a characteristic response to nitrotoluenes [18], and increased expression of transcripts coding a broad suite of elements involved in the immune response in the malaria treatment (Table 1, Fig. 3, Additional file 1: Table S6). Additionally, stressor combinations such as TNT exposure and food limitation showed transcriptional expression indicative of impaired lipid transport / metabolism causing complex effects on growth, while TNT exposure combined with malaria infection showed potential for enhanced immune activation (Fig. 3, Additional file 1: Table S6 and discussed below). In the following discussion, further attention is given to describing the *S. occidentalis* transcriptome, the mechanistic connections to both individual and combined stressors, and the implications of multiple stressor exposures in this representative lizard model species.

**Table 2:** Summary of stressor phenotypes from McFarland et al. [1] putatively matched to corresponding differentially expressed transcripts. Explanation of matches are provided in the Discussion text

Stressor Phenotype	Transcriptional			Gene
	Expression	Gene Transcript	Accession #	Symbol
Effects of TNT Exposure				
*Effects on Blood Homeostasis:*				
Decreased mean corpuscular hemoglobin concentration (MCHC)	Increased	ELG 9 homolog 3	XP-421233.2	E3LN3
Anemia (Decreased RBCs)	Increased	hemoglobin alpha, adult chain 2	NP-037228	Hba1
	Increased	hemoglobin subunit alpha-D	NP-001004375	HBAD
	Increased	erythrocyte membrane protein band 4.1-like 2	XP-002192849	
	Decreased	coagulation factor IX	NP-989674.1	F9
	Increased	cathepsin D precursor	NP-990508	CTSD
	Increased	chitotriosidase	XP-001518594	
*Energy Metabolism and Body Weight:*				
Hormesis - Increase in low TNT; decreased at high TNT	Decreased	apolipoprotein A-I precursor	NP-990856.1	APOA1
	Decreased	apolipoprotein A-V	XP-417939.2	APOA5
	Decreased	apolipoprotein B	NP-001038098.1	APOB
	Increased	chitotriosidase	XP-001518594	
	Increased	pyruvate kinase	NP-990800.1	FKM
	Increased	glucose phosphate isomerase	NP-001006128.1	GPI
	Increased	ubiquitin carboxyl-terminal esterase L1	NP-001073681.1	UCHL1
	Increased	ubiquitin specific protease 4	NP-001006134.1	USP4
	Increased	CNDP dipeptidase 2	NP-001006385.1	CNDP2
	Increased	peroxisome proliferative activated receptor y	XP-416082.2	
Effects of Malaria Infection				
*Immune Response:*				
Increased WBCs, Lymphocytes & Monocytes	Increased	major histocompatibility complex (MHC)	NP-032235.1	Mr1
Increased spleen size	Increased	CD74 molecule MHC class II invariant	NP-001001613.1	CD74
	Increased	T-cell co-stimulator	NP-001093758.1	ICOS
	Increased	linker for activation of T-cells family member 2	NP-001007483.1	LAT2
	Increased	T-cell surface glycoprotein CD3 epsilon chain precursor	NP-996787.1	CD3E
	Increased	glutathione peroxidase 1	NP-001130041.1	GPX1
	Increased	CD8 antigen alpha polypeptide	NP-990566.1	CD8A
	Increased	interferon regulatory factor 1	NP-001152868.1	Irf 1
	Increased	interferon regulatory factor 1 isoform a	XP-002188628.1	
	Increased	interferon-gamma-inducible p47 GTPase	XP-001377372.1	
	Increased	pyruvate kinase	NP-990800.1	PKM
	Increased	glucose-6-phosphate 1-dehydrogenase	NP-058702.1	G6pd
	Increased	interferon-gamma	XP-001377372.1	
	Increased	interleukin 2 receptor	NP-990197.1	BCL2A1
Effects of Food Limitation				
*Systemic Cellular Energy:*				
Decreased Body Weight	Decreased	NADH dehydrogenase: ubiquinone	NP-001006518.1	NDUFS1
Decreased inguinal fat, brain and testes weights	Decreased	short-chain acyl-CoA dehydrogenase	NP-001006193.1	ACADS
Decreased Albumin and Choleserol	Decreased	ATP synthase	XP-422453.1	ATPAF1
	Increased	peroxisome proliferator-activated receptor binding protein	XP-418125.2	MED1
	Increased	apolipoprotein A-I precursor	NP-990856.1	APOA1
*Immune Response:*				
Decreased WBCs, Lymphocytes, Eosinophils & Basophils	Generalized Responses	diminished energy and/or general stressor mediated decrements to standing immunity		
Interactive Effects -TNT and Food Limitation				
*Body Weight Change and Inguinal Fat Bodies*				
TNT-induced hormesis on body weight was abolished under food restriction.	TNT Increase (nullified)	annexin A1	NP-996789.1	ANXA1
	TNT Increase (nullified)	annexin A2	NP-990682.1	ANXA2
Reduced inguinal fat in food-restricted lizards was abolished by TNT exposure.	Decrease (enhanced)	apolipoprotein A-I precursor	NP-990856.1	APOA1
	Decrease (enhanced)	apolipoprotein B	NP-001038098.1	APOB
Interactive Effects - Malaria Infection and Food Limitation				
*WBCs /Immune Response* -				
Decreased WBCs & Lymphocytes under food limitation were	Increased (enhanced)	CD74 molecule MHC class II invariant	NP-001001613.1	CD74
increased up to *ad libitum *levels under malaria infection	Increased (enhanced)	T-cell co-stimulator	NP-001093758.1	ICOS
	Increased (enhanced)	linker for activation of T-cells family member 2	NP-001007483.1	LAT2
	Increased (enhanced)	T-cell surface glycoprotein CD3 epsilon chain precursor	NP-996787.1	CD3E
	Increased (enhanced)	CD8 antigen alpha polypeptide	NP-990566.1	CD8A
	Increased (enhanced)	interferon regulatory factor 1	NP-001152868.1	Irf 1
	Increased (enhanced)	interferon regulatory factor 1 isoform a	XP-002188628.1	
	Increased (enhanced)	interferon-gamma-inducible p47 GTPase	XP-001377372.1	
	Increased (enhanced)	interferon-gamma	XP-001377372.1	
	Increased (enhanced)	interleukin 2 receptor	NP-990197.1	BCL2A1
Interactive Effects - TNT and Malaria Infection				
*WBCs /Immune Response*				
Both TNT and Malaria caused increased WBCs	Increased (enhanced)	B-cell linker protein	NP-990239.1	BLNK
	Increased (enhanced)	T-cells family member 2	NP-001007483.1	LAT2
*Testes Size*				
TNT x Malaria reduce testes size nearly 70% - No testes expression, but systemic energy effects	Decreased	apolipoprotein A-I precursor	NP-990856.1	APOA1

### *Sceloporus occidentalis* transcriptome

Reptiles tend to be an under-represented taxonomic group in genome sequencing efforts. Transcriptome characterization for *S. occidentalis* provided 24,313 significant RefSeq (protein) annotation matches (Additional file 1: Table S3), representing a greatly expanded palette for transcriptomic investigations in this important ecological model species. The annotation matches indicated that *S. occidentalis* had the highest number of homologous transcript matches to two bird species, *Gallus gallus* (chicken) and *Taeniopygia guttata* (zebra finch, Additional file 1: Table S3). Molecular phylogeny has historically demonstrated close relationships among squamate reptiles and birds [26]. Given the intensive genome sequencing efforts for both *G. gallus* [27] and *T. glutta* [28] relative to any squamate reptiles, it is not surprising that there was high proportion of matches to these species. A primary assumption for the following discussion is ancestral orthology among gene transcripts for *S. occidentalis* and the species to which best transcript matches were identified. This assumption is best held for closely-related phylogenetic relatives of *S. occidentalis* and for genes with highly conserved functions. Overall, this transcriptome characterization provided considerable value for transcriptomic investigations in *S. occidentalis*.

### Effects of TNT exposure

#### Effects on blood homeostasis

Red blood cell lysis is a characteristic effect of TNT dosing in WFL [6] and was observed in the animals examined in the present genomics investigation [1]. Increased expression of Egl-9 family hypoxia inducible factor 3 (EGLN3, XP_421,233.2), a hypoxia inducible factor [29], was highly correlated with decreased mean corpuscular hemoglobin concentration (MCHC) in the lizards [1], Fig. 4b). Significantly increased transcriptional expression of the hemoglobin alpha, adult chain 2 (NP_037228) was observed at all TNT dosing levels regardless of the feeding treatment in Exposure 1 and in the 5 and 10 mg/L TNT doses in Exposure 1 for hemoglobin subunit alpha-D (NP_001004375, Additional file 1: Tables S4-A and S8). Functional hemoglobin is composed of two alpha and two beta hemoglobin subunits that allows efficient oxygen transport in red blood cells (RBCs) [30]. Increased hemoglobin expression is a likely response to TNT-induced decreases in hemoglobin concentrations [1] as a result of TNT-induced methemoglobinemia [31], as well as TNT-induced anemia [1] and possible hypoxia. Additionally, transcriptional expression for a gene involved in erythrocyte membrane protein band 4.1-like 2 (XP_002192849), a class of proteins involved in RBC cytoskeletal structure [32], was also increased (Additional file 1: Table S4-A). Taken together, these transcriptional observations are suggestive of increased erythropoiesis in TNT exposures in response to the TNT-induced RBC lysis and/or methemoglobinemia in the lizards [1].

Differentially expressed genes in liver were enriched in functions involved in blood coagulation and blood vessel repair (Table 1, Fig. 4a, Additional file 1: Table S6). For example, coagulation factor IX (NP_989,674.1), a blood clotting factor responsible for hemophilia B in humans [33], had decreased transcriptional expression in the 10 and 20 mg/kg/d exposures when combined with dietary restriction (Additional file 1: Table S6). Conversely, significantly increased transcriptional expression for cathepsin D precursor (NP_990,508) was observed for the 5 and 10 mg/L TNT doses in the *ad libitum* feeding group, the 5, 10 and 20 mg/L doses in the restricted diet feeding group, and in the 10 and 20 mg/L TNT doses for non-infected and malaria infected lizards from Exposure 3, respectively (Additional file 1: Table S6). Cathepsin D is a recognized proteolytic agent involved in initial wound healing responses [34]. TNT exposure has been observed to disrupt blood vessel integrity, where dermal exposure in earthworms elicited hemorrhaging [35]. The altered transcriptional expression of genes involved in coagulation and wound healing in response to TNT are indicators of potential hemostasis responses to protect RBC status where increasing expression of coagulation factor and cathepsin showed positive correlations with RBC count, hemoglobin concentration and hematocrit (Fig. 4a).

#### Energy metabolism and body weight

TNT exposures elicited complex effects on whole body weights in the WFLs causing a hormesis response [36] constituted by a biphasic dose-response of increasing body weight at low dose levels contrasted against decreased body weights at high doses in the present study and in previous TNT exposures [1, 6]. A variety of significantly enriched functions related to basal energy metabolism including carbohydrate, lipid, and protein metabolism were observed in both the Exposure 1 and Exposure 3 TNT-dosing assays (Table 1). Nitrotoluenes have been observed to initiate an adverse outcome pathway (AOP) leading to the adverse outcome (AO) of starvation-like weight loss ([18, 37], https://aopwiki.org/aops/6), which may have contributed to the weight loss in WFLs at high TNT dose levels. Impaired peroxisome proliferator-activated receptor α (PPARα) signaling represents a critical key event (KE) within the AOP causing decreased transcriptional expression of multiple genes that drive lipid catabolism and other pathways central to cellular energy regulation [37, 38]. The KEs ultimately lead to the weight loss AO, which has been observed in response to nitrotoluenes in multiple species (https://aopwiki.org/aops/6, [14, 17, 18, 37, 39]).

In accordance with the AOP, multiple PPARα-regulated transcripts (http://www.genome.jp/kegg-bin/show_pathway?hsa03320) showed dose-responsive decreases in expression in response to TNT including apolipoprotein A-I precursor (NP_990,856.1), apolipoprotein A-V (XP_417,939.2), and apolipoprotein B (NP_001038098.1; Additional file 1: Table S6 and S8). Apolipoproteins facilitate systemic lipid and cholesterol transport in support of lipid metabolism [40, 41]. As a result, decreased expression of apolipoproteins in response to TNT in the lizards is suggestive of decreased lipid transport and metabolism which could impair use of inguinal fat stores observed during dietary restriction alone [1]. Accumulation of lipids leading to fatty livers has been observed in fathead minnows exposed to 2,4-dinitrotoluene [42] and in rats exposed to various nitrotouenes [39]. Overall, the responses correspond to KEs within the weight loss AOP indicating diminished potential for energy production via lipid substrates in the TNT exposures. Further, chitotriosidase (Chit, XP_001518594) had significant positive dose-responsive increases in transcript expression in both Exposure 1 and Exposure 3 with fold changes as high as 252 at the highest TNT dose regardless of food limitation or malaria infection (Additional file 1: Table S4-A and S4-C). Dramatic increases in Chit expression have been observed in connection with lipid storage disorders and with a hematological disorder involving storage of erythrocyte membrane breakdown products [43]. Thus, Chit expression has potential value as a physiological marker for toxicological responses to TNT in WFL.

Similar to 60 d subchronic 2,6-dinitrotoluene dosing in Northern bobwhite [17] and 14 d 2,4-dinitrotoluene dosing in mice [18], TNT dosing in the WFLs elicited expression of transcripts in the glycolysis/gluconeogenesis pathway (http://www.genome.jp/kegg-bin/show_pathway?map00010) indicative of metabolic inertia toward cellular energy production versus storage. For example, pyruvate kinase (NP_990,800.1) transcript expression showed a positive dose-response relationship with TNT that was further increased by food limitation (Additional file 1: Tables S6 and S8). Pyruvate kinase is a critical negative-feedback regulator of glycolytic ATP production as well as substrate formation in the citric acid cycle [44], where increased transcriptional expression likely served as a mitigation response to low ATP concentrations and/or depleted pyruvate substrates for use in energy production. Additionally, glucose phosphate isomerase (NP_001006128.1) transcript expression showed a positive dose-response with TNT (Additional file 1: Tables S6), which is indicative of glucose processing for use as a feedstock in glycolysis [44]. These results are consistent with KEs within the weight loss AOP, where loss of energy production from lipid increases demands on alternative energy substrates, including carbohydrates.

Increased expression of gene transcripts involved in protein degradation were also observed in response to TNT in both Exposure 1 and Exposure 3 (Table 1, Additional file 1: Table S6), a response consistent with 2,6-dinitrotoluene exposures in Northern bobwhite [17]. Specifically, significant increases in ubiquitin carboxyl-terminal esterase L1 (NP_001073681.1), ubiquitin specific protease 4 (NP_001006134.1), and CNDP dipeptidase 2 (NP_001006385.1) transcript expression were observed in addition to increased expression for proteasome alpha 5 subunit, (NP_001026578.1, Additional file 1: Table S6 and S8). The proteasome is a molecular machine that catalyzes the degradation of the majority of proteins in cells [45]. Ubiquitin-protein conjugation is a critical mechanism for marking proteins for proteolysis via the proteasome [46]. Consistent with the weight loss AOP (https://aopwiki.org/aops/6, [37]), negative energy budgets due to inhibited lipid metabolism and depleted carbohydrate resources necessitates catabolism of structural protein to meet energy homeostasis. Loss of muscle mass [47] and overall body weight [18, 47] have been observed in birds and mice in response to nitrotoluene dosing, a response hypothesized and validated in mice to occur via the weight loss AOP [18]. The present observations of weight loss and concordant functional transcriptomic profile, strongly suggest that the weight loss of WFLs in response to TNT may occur via this AOP.

#### Hormesis response – Body weight

The hormesis response of increased body weight relative to controls in low-dose TNT exposures has the potential to be explained within the mechanistic components of the AOP for weight loss. Specifically, qPCR validated increases in peroxisome proliferative activated receptor gamma (PPARγ, XP_416,082.2) were observed at low and intermediate concentrations of TNT (Additional file 1: Tables S6 and S8). Increased transcriptional expression of PPARγ in mice has been observed as a compensatory response to PPARα knockout and in response to 2,4-dinitrotoluene dosing [18]. Thus, hormesis in the present study may result from compensatory increases in cellular energy production initiated through stimulation of alternative energy homeostatic signaling pathways such as increase PPARγ signaling.

### Effects of malaria infection

#### Immune response

To understand the immune response to malaria infection in WFL, it is important to have a basic understanding of the *Plasmodium* life cycle in the host. The phlebotomine sand fly (*Lutzomyia vexator*) is the typical vector which transmits the malarial disease agent *P. mexicanum* into WFL during blood feedings [48]. The plasmodium life cycle in mammalian hosts is succinctly summarized [49]; the majority of life cycle processes are analogous in lizards [50]. Briefly, the sporozoite generation of *Plasmodium* serves as the virulent agent transmitted to the vertebrate host which migrates into the blood where it ultimately infects hepatocytes. The sporozoites differentiate into mature liver-stage schizonts with thousands of uninucleated merozoites which are released into the bloodstream upon maturation and hepatocyte rupture. The merozoites infect developing red blood cells (reticulocytes), thus beginning the blood-stage infection. They mature into erythrocytic trophozoites that rupture red blood cells and release additional merozoites, inducing cyclic reticulocyte infection and symptoms of severe malaria in the host [49].

In the present study, the “immune response” to the malaria infection treatment was the most significantly enriched annotation cluster within this entire study (Table 1, Exposure 2, Additional file 1: Table S5) where increased transcriptional expression was predominant (Fig. 3, Additional file 1: Table S6) and qPCR validated (Additional file 1: Table S9). Given the complexity of *Plasmodium* life cycle progression in the vertebrate host, it is not surprising that a variety of immune response processes are deployed to combat infection at each developmental stage [49], many of which were reflected as corresponding changes in transcriptional expression in WFL (Table 1).

Cellular-level immune responses indicative of malarial liver-stage hepatocyte infection characteristic in humans, including induction of the major histocompatibility complex (MHC) response [49] had increased transcriptional expression in the lizards (Additional file 1: Table S6). This included increased transcriptional expression of the CD74 molecule MHC class II invariant (NP_001001613.1, Additional file 1: Table S6) which in mammalian species enables presentation of foreign peptides on cell surfaces to flag infection for T-cell response [51, 52] and demonstrated positive correlations with monocyte counts (Fig. 4d). Increased expression of genes involved in T-cell responses were also observed in the malaria infected lizards, including inducible T-cell co-stimulator (NP_001093758.1), a linker for activation of T-cells family member 2 (NP_001007483.1) and T-cell surface glycoprotein CD3 epsilon chain precursor (NP_996,787.1), each of which represent critical elements in T-cell activation and proliferation in mammalian species (Additional file 1: Table S6, [53, 54]). Additionally, co-expression analysis identified a correlation between glutathione peroxidase 1 (GPX1, NP_001130041.1) and WBC concentrations (Additional file 1: Table S7) where GPX1 and additional glutathione-related transcripts had increased expression in response to malaria (Additional file 1: Table S4-B and S4-C). This response is indicative of lymphocyte proliferation in humans [55]. Taken together, these transcriptomic results correspond with the increased lymphocyte counts observed in the infected lizards (Table 1, [1]). In concert with T-cell activation, increased transcriptional expression of CD8 antigen alpha polypeptide (NP_990,566.1, Additional file 1: Table S6) is indicative of an innate immune response in humans to liver-stage infection where CD8+ T-cells kill hepatic schizonts [56].

Transcriptional responses characteristic of blood-stage malarial infection were also observed, including dramatically increased transcriptional expression of interferon regulatory factor 1 (NP_001152868.1), interferon regulatory factor 1 isoform a (XP_002188628.1) and interferon-gamma-inducible p47 GTPase (XP_001377372.1, Additional file 1: Table S4-B). In mammalian malaria infection, induction of interferon expression has been demonstrated to enhance humoral immunity increasing antibody and cytokine production [57], responses indicative of an immune system offensive against the blood-stage merozoite infection of reticulocytes [49]. In mammalian malaria, both pyruvate kinase and glucose-6-phosphate gene variants have been implicated in host resistance to *Plasmodium* infection by protecting reticulocytes / erythrocytes [49, 58, 59]. Malaria infected WFL demonstrated increased expression of pyruvate kinase (NP_990,800.1) and glucose-6-phosphate 1-dehydrogenase (NP_058702.1, Additional file 1: Tables S4-B and S6), perhaps in response to matriculated malaria infection targeting erythrocytes. Finally, the increased expression of interferon-gamma (XP_001377372.1, Additional file 1: Table S4-B) and interleukin 2 receptor (NP_990,197.1, Additional file 1: Table S6 and S8) represent potentially important immune responses given that these are critical elements of the resting memory of malaria-exposure in memory T-cells in humans [60].

### Effects of food limitation

#### Systemic cellular energy

Food limitation represents a generalized stressor that can affect practically all physiological processes. The general nature of the stressor may have contributed to the observation of minimal transcriptomic-expression responses conserved among the repeated food limitation exposures (Exposures 1 and 2, Fig. 2b). At the functional level, significant enrichment of transcripts involved in mitochondrial processes were conserved across the food limitation treatments in Exposure 1 and Exposure 2 (Table 1), where expression was predominantly decreased (Additional file 1: Table S6). Decreased transcriptional expression for components of NADH dehydrogenase: ubiquinone, (NP_001006518.1), short-chain acyl-CoA dehydrogenase (NP_001006193.1), and ATP synthase (XP_422,453.1, NP_001091003.1) each of which is crucial for electron transport chain-mediated ATP production [61, 62], presumably reflects the decreased potential for cellular energy production under food limitation. Decreased food resources increase the need to use stored energy reserves, which was reflected by increased expression of peroxisome proliferator-activated receptor binding protein (PPAR BP, XP_418,125.2, Additional file 1: Table S6) where PPARs, especially PPARγ, are recognized to mobilize stored-lipid metabolism [38]. Correspondingly, apolipoprotein A-I precursor (NP_990,856.1), which facilitates systemic lipid and cholesterol transport in support of lipid metabolism [40, 41] also showed increased expression in response to food restriction (Additional file 1: Table S6 and S8). Resultant phenotypic observation of decreased inguinal fat and decreased body-weight loss (Table 1, [1]) indicate that, as expected, diet restriction negatively affected the overall systemic energy budget of WFL.

#### Immune response

Human epidemiological studies have demonstrated that starvation diminishes effective immune responses thus increasing disease susceptibility [63]. In the present study, food restriction was sufficient to negatively impact the WFL systemic energy budget, and thus the energetically expensive basal immune response [9] was diminished as evidenced by significantly reduced standing stocks of WBCs including lymphocytes, eosinophils and basophils (Table 1, [1]). Experimental investigations of malnutrition in rats have also demonstrated decreased blood levels of lymphocytes and granulocytes [8]. Likewise, calorie deficiency in conjunction with extreme exercise and sleep deprivation has been observed to decrease lymphocyte numbers for all major WBC subgroups including CD4 T-cells, CD8 T-cells, B cells and natural killer cells in humans [64]. In humans, general stress responses from fasting have also been shown to stimulate corticosteroid production which had an inverse correlation with CD4 cell numbers in blood [65], a characteristic response of lymphocytes following corticosteroid induction [66]. In the present study, food restriction did not elicit specific changes in transcriptional expression of immune-related genes in WFL, thus the phenotypic effects of decreased WBC counts are hypothesized to result from diminished energy allocations and/or general-stressor mediated decrements to standing immunity.

### Interactive effects of TNT and food limitation

#### Body weight change and inguinal fat bodies

Given the effects of TNT on systemic energy metabolism (revisit weight loss AOP discussed above), interactive effects between TNT and food limitation treatments (Table 1, Fig. 4c, [1]) were not surprising. For example, the observed hormesis response of significantly increased body weight caused by exposure to lowest dose of TNT was eliminated when food was limited. Correspondingly, the reduced inguinal fat weights measured in food-limited lizards was abolished by TNT exposure [1], likely as a function of TNT-induced inhibition of lipid mobilization and metabolism (see discussion for “Effects of TNT Exposure” above). Transcriptomics results indicated that the significantly increased transcriptional expression of annexin A1 (NP_996,789.1) and annexin A2 (NP_990,682.1) in response to TNT were also abolished under restricted feeding (Additional file 1: Table S6 and S8). Annexins have been identified to reduce the rate of lateral lipid diffusion [67], and also influence lipid-mediated signaling pathways by affecting the distribution and activity of lipid metabolizing enzymes [68]. As discussed above, apolipoproteins additionally facilitate systemic lipid transport in support of lipid metabolism [40, 41]. Already-decreased transcriptional expression for apolipoprotein A-I precursor (NP_990,856.1) and apolipoprotein B (NP_001038098.1) were exacerbated by restricted feeding at the 5 mg/kg/d (Additional file 1: Table S6). The combination of transcriptomic and inguinal fat body responses following combined TNT exposure and food limitation (Fig. 4c) strongly suggest interactive effects on lipid metabolism that results in fat immobilization and systemic energy budget decreases, which impair lizard growth.

### Interactive effects of malaria infection and food limitation

#### WBCs / immune response

The standing stocks of lymphocytes and overall white blood cells (WBCs) were significantly diminished in the lizards when food was restricted [1]. However, when food-restricted lizards were challenged with malaria infection, WBCs and leukocytes increased up to levels observed in malaria infected lizards that were fed *ad libitum*[1]. Correspondingly, transcriptional expression for WBC activation in response to malaria infection was markedly increased in food-limited versus well-fed lizards (Fig. 3). Given an already diminished stock of WBCs under food limitation, the transcriptional results suggest an enhanced-scale WBC activation response to combat the malaria infection relative to well-fed lizards. Overall, the transcriptional expression for the various immune-related annotation clusters enriched in response to malaria infection were increased across the board when comparing restricted feeding versus well-fed animals (Additional file 1: Table S6). Thus, in addition to WBC activation, the full suite of humoral immune responses to malaria (discussed in “Effects of Malaria Infection” section above) also appeared to be enhanced in the restricted feeding treatment. The observations suggest that food limitation, at least at the levels tested, may not impair the WFLs’ capacity to launch a robust immune response to malaria infection.

### Interactive effects of TNT and malaria infection

#### WBCs / immune response

Interestingly, exposure to TNT in WFLs caused increases in WBC concentrations, which was a similar response to lizards infected with malaria [1]. Transcriptional expression also increased in both TNT and malaria exposures for immune responses, including increased expression for B-cell linker protein (NP_990,239.1, Additional file 1: Table S6), a functional component of B-cell activation [69]. To some degree, the results suggest an enhanced immune activation caused by TNT exposure that may have had a complimentary effect toward the immune response to malarial infection. However, some interactive effects among TNT exposures and malaria infection were observed that may have impaired the immune response. For example, the linker for activation of T-cells family member 2 (NP_001007483.1), which is a component of T-cell signaling pathways [70] had increased expression in malaria infected lizards and positive dose-response relationships with TNT exposure. However, when combined, TNT had an antagonistic effect reducing expression (Additional file 1: Table S9). Overall, both malaria infection and TNT tended to increase transcriptional expression of immune-related genes and increase overall WBC concentrations in blood. Some unique and interactive effects among the stressors were observed that likely lead to changes in immune profile, such as the observed decreases in heterophil to lymphocyte ratios in the combined exposures [1]. The effects of combined stressors are likely even more complex in environmental exposures, but the present results indicate that TNT, even at relatively high exposure concentrations, may not impair the immune response to malaria in lizard.

#### Testes size

TNT and malaria infection each individually caused reduced testes size in the lizards. The combined stressors caused additive effects at the highest TNT exposure concentration, reducing testes weights by nearly 70% [1]. The present genomics investigation assayed liver tissue, so only peripheral inferences can be drawn regarding this observation. However, impaired bioenergetics in both TNT and malaria infections (discussed in previous sections) are likely contributors. For example, a correlation was identified between decreased transcriptional expression of apolipoprotein A-I precursor (NP_990,856.1), an indicator of diminished energy from lipid metabolism, and decreasing testes weights in the TNT exposure (Additional file 1: Table S8). Additionally, an inverse correlation in transcriptional expression for genes involved in immune-response activation and testes weights was observed (Fig. 4e). Malaria infection induced a dramatic immune response in the lizards, which is a bioenergetically-costly process and likely deferred energy from maintaining reproductive tissues. Neither functional enrichment nor correlation analysis identified androgenic targets affected in TNT exposure or malaria infection. However, transcriptional expression in liver may not reflect androgenic conditions in testes. Malaria infection can also reduce reproductive fitness in WFLs by diminishing courtship behaviors in males [4, 5]. Luckily, the additive effects of malaria infection and TNT dosing on testes were only observed at very high TNT exposure concentrations (40 mg/kg/d), which are unlikely in the field. However, TNT x malaria effects on mating behaviors may still be a concern at more environmentally relevant TNT exposure levels and should be further tested.

## Conclusions

Stressors representative of climate change, habitat encroachment and chemical contamination individually elicited potential adverse effects in WFLs. The global transcriptomic expression analyses provided insights into the molecular processes underlying these effects (Tables 1 and 2). From a transcriptomic perspective, responses to the combined stressors tended to operate independently of one another when molecular targets among stressors did not overlap. On the other hand, when stressors had common molecular targets, there was greater potential for additive or interactive effects. Specifically, both TNT and food limitation each impaired systemic energy production, leading to interactive effects on lipid metabolism and overall growth. Additionally, TNT and malaria infection each individually increased immune responses in the lizards, where the combined stressors likely complimented immune activation against malaria infection. Lastly, although food restriction impaired immunity, a compensatory immune activation at the transcriptomic level was observed resulting in a fully competent immune response to malaria. Additional investigations are needed to fully uncover the mechanisms underlying additive impacts of TNT and malaria on testes size. Overall, results thus far suggest remarkable resilience of WFLs to multiple-stressor exposures.

## Additional files

Additional file 1: Tables. Include additional results in support of the main manuscript and are provided in the file. (XLSX 2291 kb)

Additional file 2: Figures. Include additional graphics in support of the main manuscript and are provided in the file. (DOCX 2097 kb)

## Abbreviations

AO: Adverse outcome
AOP: Adverse outcome pathway
blastx: Basic local alignment search tool - protein
bp: Base-pair
cDNA: Complimentary DNA
contigs: Contiguous sequences
DAVID: Database for annotation, visualization and integrated discovery
DNA: Deoxyribonucleic acid
DoD: Department of Defense
EASE: Expression Analysis Systematic Explorer
EBI: European Bioinformatics Institute
emPCR: Emulsified in oil for polymerase chain reaction
ESTs: Expressed sequence tags
GEO: Gene Expression Omnibus website
KE: Key event
MC: Munitions constituent
MCHC: Mean corpuscular hemoglobin concentration
MHC: Major histocompatibility complex
NCBI: National Center for Biotechnology Information
PBS: Portable Batch System
PCR: Polymerase chain reaction
RBCs: Red blood cells
RDX: 1,3,5-trinitro-1,3,5-triazine
RIN: RNA integrity number
RNA: Ribonucleic Acid
rRNA: Ribosomal RNA
RT-qPCR: Reverse-transcriptase quantitative polymerase chain reaction
singlets: Single sequences
ssDNA: Single stranded DNA
TGICL: The Gene Indices Clustering Tools
TNT: 2,4,6-trinitrotoluene
USAPHC: U.S. Army Public Health Center
WBC: White blood cell
WFL, *Sceloporus occidentalis*: Western fence lizard
WGCNA: Weighted gene co-expression network analysis
