# Supplementary material for: Multiple environmental stressors induce complex transcriptomic responses indicative of phenotypic outcomes in Western fence lizard
Source: BMC Genomics. 2018 Dec 5;19:877. doi: 10.1186/s12864-018-5270-0 (PMC6282355; doi:10.1186/s12864-018-5270-0)
Supplement: Supplementary file 2 — Figures. Include additional graphics in support of the main manuscript and are provided in the file. (DOCX 2097 kb) [file 12864_2018_5270_MOESM2_ESM.docx]

**Supplementary Figures**

**Title: Multiple Environmental Stressors Induce Complex Transcriptomic Responses Indicative of Phenotypic Outcomes in Western Fence Lizard**

**Authors:** Kurt A. Gust^a*^, Vijender Chaitankar^b^, Preetam Ghosh^c^, Mitchell S. Wilbanks^a^ Xianfeng Chen^d^, Natalie D. Barker^e^, Don Pham^f,g^, Leona D. Scanlan^f,h^, Arun Rawat^i^, Larry G. Talent^j^, Michael J. Quinn Jr.^k^, Christopher D. Vulpe^l^, Mohamed O. Elasri^m^, Mark S. Johnson^k^, Edward J. Perkins^a^, Craig A. McFarland^k^

^a^U.S. Army Engineer Research and Development Center, Environmental Laboratory, Vicksburg, MS, USA 39180. [kurt.a.gust@usace.army.mil](mailto:kurt.a.gust@usace.army.mil), [mitchell.s.wilbanks@usace.army.mil](mailto:mitchell.s.wilbanks@usace.army.mil), [edward.j.perkins@erdc.dren.mil](mailto:edward.j.perkins@erdc.dren.mil)

^b^National Institute of Health - National Heart, Lung, and Blood Institute, Bethesda, MD, USA 20892. [vijender.chaitankar@nih.gov](mailto:vijender.chaitankar@nih.gov).

^c^Virginia Commonwealth University, School of Engineering, Richmond, VA, USA 23284. [pghosh@vcu.edu](mailto:pghosh@vcu.edu)

^d^IFXworks LLC, 2915 Columbia Pike, Arlington, VA 22204, USA. [xianfeng.chen@ifxworks.com](mailto:xianfeng.chen@ifxworks.com)

^e^Bennett Aerospace, Cary, NC 27511, USA. [natalie.d.barker@usace.army.mil](mailto:natalie.d.barker@usace.army.mil)

^f^Department of Nutritional Sciences and Toxicology, University of California Berkeley, Berkeley, California 94720

^g^(Current Affiliation) Carlsbad Unified School District, Carlsbad, CA, USA 92009. [donquypham@gmail.com](mailto:donquypham@gmail.com)

^h^(Current Affiliation) California Environmental Protection Agency, Department of Pesticide Regulation, Sacramento, CA, USA 95812. Scanlan.Leona@gmail.com

^i^Sidra Medicine, Education City (North Campus), Doha, Qatar 26999. [rawat.arun@gmail.com](mailto:rawat.arun@gmail.com)

^j^Oklahoma State University, Department of Natural Resource Ecology and Management, Stillwater, OK, USA 74078. [larry.talent@okstate.edu](mailto:larry.talent@okstate.edu)

^k^U.S. Army Public Health Center, Aberdeen Proving Ground, MD, USA 21010. [michael.j.quinn104.civ@mail.mil](mailto:michael.j.quinn104.civ@mail.mil), [mark.s.johnson.civ@mail.mil](mailto:mark.s.johnson.civ@mail.mil), [craig.a.mcfarland.civ@mail.mil](mailto:craig.a.mcfarland.civ@mail.mil)

^l^University of Florida, College of Veterinary Medicine, Gainesville, FL, USA 32610. [cvulpe@ufl.edu](mailto:cvulpe@ufl.edu)

^m^University of Southern Mississippi, Department of Biological Sciences, Hattiesburg, MS, USA 39406-5018. [mohamed.elasri@usm.edu](mailto:mohamed.elasri@usm.edu)

*Corresponding Author: [kurt.a.gust@usace.army.mil](mailto:kurt.a.gust@usace.army.mil)

**Supplementary Figure S1.** Principal component analysis (PCA) of differentially expressed transcripts for exposures 1-3. PCA plots were developed for each primary stressor in each exposure.

**Supplemental Figure S2** (page 1 of 3). Heat maps and hierarchical clustering analyses for transcripts having significant differential expression in the treatment-interaction tests (1. TNT x Food Limitation, 2. Malaria x Food Limitation, and the 3. TNT x Malaria) identified by 2-way ANOVA.

**Supplemental Figure S2** cont. (page 2 of 3)

**Supplemental Figure S2** cont. (page 3 of 3)
